# Children with Congenital Hypothyroidism Have Similar Neuroradiological Abnormal Findings as Healthy Ones

**DOI:** 10.1155/2013/194918

**Published:** 2013-10-03

**Authors:** Marianna Rachmiel, Susanne Blaser, Elysa Widjaja, Joanne Rovet

**Affiliations:** ^1^Pediatric Endocrinology Service, Division of Pediatric, Assaf Harofeh Medical Center, Sackler School of Medicine, Tel Aviv University, Zerifin 70300, Israel; ^2^Division of Endocrinology, The Hospital for Sick Children, 555 University Avenue, Toronto, ON, Canada M5G 1X8; ^3^Department of Diagnostic Imaging, The Hospital for Sick Children, 555 University Avenue, Toronto, ON, Canada M5G 1X8; ^4^Department of Psychology and Pediatrics, The Hospital for Sick Children, University of Toronto, 555 University Avenue, Toronto, ON, Canada M5G 1X8; ^5^Neuroscience and Mental Health Program, The Hospital for Sick Children, 555 University Avenue, Toronto, ON, Canada M5G 1X8

## Abstract

*Objective*. To assess the neuroradiological findings of children with congenital hypothyroidism (CHT) compared to healthy controls (HC). *Patients and Methods*. Thirty children with CHT, mean age 12.5 ± 1.6 years, 14 (44.8%) males, were compared with 38 HC mean age 11.7 ± 1.7 years, 16 (45.7%) males. Clinical data were collected from medical charts and questionnaires seeking information on family history, birth and perinatal period events, medications, and overall health history. Neurocognitive function was assessed for global intelligence, visual and verbal memory, and executive functioning using standardized tests. Neuroimaging was performed using 1.5 T magnetic resonance imaging and assessed by two pediatric radiologists. *Results*. Children with CHT had a similar proportion of incidental findings as did the children in the HC group, at 43.3% and 39.5%, respectively, *P* = 0.69. Abnormalities of the sellar region were reported in 13.3% of CHT group and 7.9% of HC group, *P* = 0.46. Other incidental findings included cerebellar ectopia, choroidal fissure and pineal cysts, and multiple increased signal intensity foci. Neuroradiological findings were not associated with clinical and neurocognitive abnormalities. *Conclusion*. Neuroimaging of children with CHT demonstrated a similar incidence of structural abnormalities as in the healthy population. There is no association between those findings and neurocognitive function.

## 1. Introduction

Children with congenital hypothyroidism (CHT) are known to have neurocognitive impairments of varying degrees, especially on measures of learning and memory [1–3]. Although their performance typically falls within the normal range, they score significantly below controls on word-pair and object-location recall tasks [4]. These abnormalities have been linked to structural and functional brain abnormalities. Our group recently reported that CHT subjects had smaller hippocampal volumes, without the expected volume increment with age [5, 6]. However, there are no data on the neuroradiological structure in the CHT population; typically, incidental abnormal findings on neuroimaging studies are reported in 10.9–21% of healthy volunteers undergoing magnetic resonance imaging (MRI) [7, 8]. The primary aim of our study was to characterize the neuroimaging abnormalities in children with CHT in comparison with age-matched healthy controls (HC). A secondary aim was to correlate these findings with neurocognitive assessment and indices of initial hypothyroidism severity and management.

## 2. Methods

### 2.1. Design and Study Population

This is a case control study in which data were extracted from two cohorts with similar population and task requirements. Written informed consent was obtained from the parents or guardians of the children. The studies were approved by the Research Ethics Board of The Hospital for Sick Children.

Participants with CHT were recruited from the Endocrine Clinic at The Hospital for Sick Children, Toronto, Canada. Age- and gender-matched healthy controls were recruited by advertisements in schools, community organizations, and hospital area. Exclusion criteria were any perinatal event, systemic, neurologic, developmental, or psychiatric condition that could confound the neuroimaging or neuropsychological findings. Subjects were also excluded if they had implanted metal objects or any other condition in which MRI is contraindicated. 

### 2.2. Data Collection

Parents completed a questionnaire regarding family history, birth and perinatal period, medications and health history, pregnancy, medical history, and early development. Charts of children with CHT were reviewed for data at diagnosis and at tests performance. 

### 2.3. Neuroimaging

MRI was performed in the Diagnostic Imaging Unit at The Hospital for Sick Children, Toronto, Canada, using a 1.5 T General Electric Excite Twin Speed scanner (Milwaukee, WI). Multiplanar multisequence images were performed through the brain in sagital T1, axial T2, and FLAIR and coronal T2, without contrast material. Images were acquired using two protocols of similar resolution: a volumetric gradient-echoT1-weighted sagittal series (TR: 8.448 msec; TE: 4.2 msec; flip angle: 15°; field of view of 220, slice thickness of 1 mm, no gap), a coronal T2-weighted sequence (TR: 4000 msec; TE: 106.944 msec; flip angle: 90°; field of view of 165, slice thickness of 5 mm), and axial FLAIR sequence (TR: 9000 msec; TE: 164. msec; flip angle: 90°; inversion time: 2250 msc, field of view of 200, slice thickness of 5 mm), and a volumetric fast gradient-echo T1-weighted axial series (TR: 10.06 msec; TE: 4.2 msec; flip angle: 20°; field of view of 180, slice thickness of 1.5 mm, no gap), sagittal T1-weighted FLAIR sequence (TR: 2162.82 msec; TE: 9.048 msec; flip angle: 90°; inversion time: 750 msc, field of view of 200, slice thickness of 5 mm), coronal T2-weighted sequence (TR: 4000 msec; TE: 88.488 msec; flip angle: 90°; field of view of 220, slice thickness of 5 mm), and axial FLAIR sequence (TR: 9002 msec; TE: 153.894 msec; flip angle: 90°; inversion time: 2200 msc, field of view of 200, slice thickness of 5 mm). All MRI studies were reviewed by two certified neuroradiologists blinded to participant group. Final report was derived by consensus. 

### 2.4. Neuropsychological Assessment

Intelligence was assessed using the Wechsler Abbreviated Scale of Intelligence (WASI) based on vocabulary and matrix reasoning subtests. Verbal memory was assessed with the Children's Memory Scale Stories subtest and visuospatial memory with the Rey-Osterrieth Complex Figure task. Executive functioning was assessed using the Behavioral Rating Inventory of Executive Functions (BRIEF). 

### 2.5. Statistical Analysis

Statistical analysis was performed using the statistical package SPSS, version 18.0.0. The Chi-squared test (*χ* ^2^) or Fisher's exact test was used to evaluate differences in the categorial parameters between the HC and CHT groups. *t*-test was used for the evaluation of differences in quantitative variables. One = way ANOVA was performed to assess possible association between neuroradiological findings and neuropsychological tests and data regarding neonatal period. *P* < 0.05 was considered significant.

## 3. Results

The CHT group included 30 participants, 14 males (46.7%), mean age 12.52 ± 1.55 years (range 10–15 years). The HC group included 38 participants, 16 males (45.7%), mean age 11.71 ± 1.72 years, (range 9–14 years). The cause of hypothyroidism was thyroid dysgenesis in 66%; 10% had dyshormonogenesis, and the cause in the rest was unknown. L-thyroxine treatment was initiated within 14.32 ± 11.92 days, median 12 days (65 in one child) with an average dose of 10.15 ± 1.47 mcg/kg/d and with TSH normalization by 3.17 ± 2.24 months of age. Normalization occurred probably earlier, that is, the level reported in the first clinic visit. Eighteen of the CHT group (62.07%) and 18 of the HC group (47.4%) reported a variety of nonneurological conditions, including mild asthma, recurrent ear infections, allergy, and uneventful operations. None had symptoms of headaches, seizures, or syncope. Four (13.3%) of the CHT group were diagnosed with an attention deficit disorder (ADHD); of these, two had normal MRI, one had tonsillar ectopia of 2.1 mm, and one had partial empty sella. Thirteen participants of the CHT group (43.3%) and 15 participants of the HC group (39.5%) had abnormal findings on neuroimaging, with no statistical difference, *P* = 0.689. The characteristics of the abnormal neuroimaging findings are described in Table 1. Abnormalities at the sella region measured according to volumetric norms for gender and age were reported in 7 of the participants, 4 with CHT (13.3%) and 3 with HC (7.9%), *P* = 0.456. The description of lesions is presented in Table 2 according to the clinical and neurocognitive characteristics of the participants. None had abnormal growth or additional endocrine abnormalities at the time of assessment. Three clinically asymptomatic children in the HC group presented with significant abnormalities despite normal neurocognitive studies: one had a 16 × 23 mm calcar avis region cyst, one had hypoplastic corpus callosum with pericallosal lipoma, and one had a wedge-shaped area in the left cerebellar hemisphere in the PICA vascular territory, with encephalomalacia. All were referred for further evaluation. No significant association was demonstrated between the neuroimaging findings and the neurocognitive assessment in either group. No significant association was detected between the cause of hypothyroidism, age of thyroid function stabilization, and neuroimaging findings.

## 4. Discussion

This is the first study to compare the frequency and severity of incidental structural neuroimaging findings of children with CHT and healthy controls. Forty-three percent of pediatric CHT patients had abnormal asymptomatic incidental findings that were unrelated to timing and dose of hormonal replacement management or to CHT etiology. This prevalence was similar to a matched healthy population, derived from the same geographical area and reviewed similarly at the same time period. In both groups, no association was observed between the findings and neuropsychological testing. The only previous report of neuroimaging in the population of children with CHT was from the same population, relating only to their reduced hippocampal volumes, particularly on the left side [6]. This report adds important information regarding the cerebral structure, including white matter, cerebellar area, and sellar region in the population of children with CHT, reinforcing the information that the only difference from healthy pediatric population is functional and volumetric at the hippocampal area. Our findings demonstrate a higher prevalence of incidental findings in both groups than reported in healthy population [7–12]. However, previously reported groups have been of wide age distribution involving children and adults and included neuroimaging studies from both healthy controls and from patients followed at neurology clinics [9, 10]. Reports of incidental neuroimaging findings are scarce in the pediatric population. The prevalence of incidental findings in pediatric cohorts imaged due to headache, epilepsy, ADHD, and migraines ranges from 20% to 51% [11, 12], 21% abnormalities in healthy volunteers aged 4–18 [7], 10.9% in ages of 5–8 [8], and 6.6% in a 5–14-year population of sickle cell disease [13]. Abnormal findings in those studies included Chiari I malformations, nonspecific white matter abnormalities, venous angiomas, arachnoid cysts, enlarged Virchow-Robin spaces, pineal cysts, and periventricular leukomalacia, similar to the findings we detected, but none reported abnormalities at the sella area. However, it is unclear if the sella area was interrogated for size in those studies. Abnormalities at the sella area, such as partially empty sella and hypoplastic pituitary gland, were seen in 13.3% of our CHT population and 7.9% of our HC population and were unrelated to the neurocognitive abilities or gender. Takanashi et al. [14] reported prevalence of 1.1% in a pediatric population attending neurology clinics, in association with hypothalamic pituitary disorders. Foresti et al. [15] detected empty sella in 28% of a population younger than 40 years, who underwent MRI of the brain for a variety of conditions not related to pathology of the sellar or juxtasellar regions. Łacka et al. [16] assessed MRI results from 21 adults with CHT and reported abnormalities at the sella area in more than 50%, with 24% of the group having a partially empty sella or hypoplasia and 35% of hyperplasia of the pituitary gland; however, most patients had significantly abnormal TSH levels. These results were explained in terms of instability of management; furthermore, they had no control group.

The fact that our study population had a higher prevalence of incidental abnormalities than in previous reports, especially in the sellar region, may be related to the highly trained personnel in pediatric neuroradiology, intentional volumetric measurements of pituitary and tonsillar area according to age and gender normative values, and higher resolution of MRI (1.5 Tesla compared to 0.5 Tesla). The relatively high incidence of findings in both populations, with no clinical relevance, probably indicates that those findings are clinically insignificant and should not be considered as pathological. 

The study strengths include the highly homogenous groups that are comparable in age distribution, gender, and geographical location, independent blinded assessment by same personal team of clinical data, neuroradiology and neurocognitive studies, and usage of same scanner for all participants. Study limitations include our relatively small population size, and despite the narrow age range of subjects, there was no data of pubertal stage at the MRI performance. Therefore, we could not assess possible enlargement of the pituitary gland.

## 5. Conclusions

 This is the first study to present information regarding the overall neuroimaging findings in a pediatric population with CHT, compared to a similar healthy population. We found no specific structural abnormalities associated with CHT, and those that were identified were unrelated to indices of early disease severity or neurocognitive outcome. This study adds to the recently described data regarding only the hippocampus area. The role of sellar abnormalities in the pediatric population should be further investigated, since we found a relatively high incidence of these lesions in both CHT and HC populations suggesting that this is a novel observation requiring further investigation.

## Figures and Tables

**Table 1:** Distribution of abnormal findings detected on MRI according to study group.

Findings on MRI	HC	CHT
Participants	38	30
Participants with incidental findings (%)	15 (39.5)	13 (43.3)
Incidental findings		
(i) Prominent VR perivascular spaces	3 (7.9)	2 (6.7)
(ii) Hypoplastic corpus callosum	1 (2.6)	0
(iii) Mild cerebellar ectopia (2.1–3.5 cm)	3 (7.9)	4 (13.3)
(iv) Calcar avis cystic structure	1 (2.6)	0
(v) Choroidal cyst	3 (7.9)	2 (6.7)
(vi) Abnormalities in sella region	3 (7.9)	4 (13.3)
(vii) Pineal cyst (<1 cm)	2 (5.2)	0
(viii) Encephalomalacia from remote ischemic event	1 (2.6)	0
(ix) Foci of increased signal intensity in the cerebral white matter on T2 (1–17 mm)	1 (2.6)	2 (6.7)

There are 32 lesions among 28 participants with abnormalities, since 4 participants had more than one abnormal finding on MRI.

**Table 2:** Characteristics of participants with incidental abnormal findings at the sella region.

Group	Gender	Age	Cause of CHT and treatment age	Finding	Neurocognitive outcome
CHT	F	10	Agenesis, day 11	Hypoplastic gland and choroidal fissure cyst 17 × 13 mm	Abnormal attention and memory, ADHD
CHT	F	13	Unknown, day 9	Persistent craniopharyngeal canal	No cognitive difficulties, weak reading
CHT	M	10	Lingual, day 13	Partial empty sella	No problems
CHT	M	14	Lingual, day 65	Partial empty sella and choroidal fissure cyst 2 mm	Abnormal attention, executive processing, and everyday memory, ADHD
HC	M	14	NA	Hypoplastic gland	Nonspecific mild behavioral abnormalities
HC	M	11	NA	Partial empty sella	Abnormal executive function
HC	M	10	NA	A small focal lesion in the sella region, pars intermedia cyst	No problems

ADHD: attention deficit and hyperactivity disorder.

## Abbreviations

CHT: Congenital hypothyroidism
HC: Healthy controls
MRI: Magnetic resonance imaging.
